# Association between educational level and postoperative delirium in older patients undergoing abdominal surgery: a two-sample cohort study

**DOI:** 10.3389/fmed.2025.1581503

**Published:** 2025-06-13

**Authors:** Mingju Xiang, Jie Liu, Jing Wang, Feng Li, Tingting Fan, Jia Tang

**Affiliations:** Department of Anesthesiology, The Second Affiliated Hospital of Chongqing Medical University, Chongqing, China

**Keywords:** older patients, postoperative delirium, abdominal surgery, risk factors, cognitive reserve

## Abstract

**Background:**

Postoperative delirium (POD) is a critical complication in older patients following abdominal surgery, significantly contributing to delayed recovery and prolonged hospital stays. Understanding the risk factors associated with POD is essential for developing effective prevention and intervention strategies. This study investigates the potential impact of educational attainment on the incidence of delirium in this patient population.

**Methods:**

This study utilized a two-sample cohort design to collect demographic and educational attainment, and clinical data, including, from older patients undergoing abdominal surgery. The assessment of delirium during the recovery phase was conducted using the Confusion Assessment Method for the Intensive Care Unit (CAM-ICU) and the 3-Minute Diagnostic Interview for Confusion Assessment Method within the first three postoperative days. In the exploratory cohort, the relationship between education and postoperative delirium was determined by univariate analysis, followed by multivariate logistic regression to determine that education was an independent predictor. The identified risk factors were subsequently validated in an independent validation cohort to ensure robustness and generalizability.

**Results:**

The exploratory cohort consisted of 342 cases, while the validation cohort included 150 cases. Exploratory cohort regression analysis identified lower educational attainment and procedures or anesthesia lasting longer than 4 h as independent risk factors for POD. Anesthesia time of more than 4 h was also an independent risk factor for delirium during resuscitation.

**Conclusion:**

Lower educational attainment is significantly related to an increased chance of POD in older adults undergoing abdominal procedures. These findings suggest that preoperative assessments should incorporate educational level as a potential risk factor, providing a basis for targeted prevention and intervention strategies to mitigate POD.

## 1 Introduction

Abdominal surgery, which includes procedures involving the hepatobiliary, pancreatic, gastrointestinal, urological, and gynecological systems (1), is a surgical intervention frequently executed in clinical practice, particularly among the older population. Postoperative delirium (POD) (2), an acute and transient neurological disorder characterized by impaired attention, altered cognition, and fluctuating consciousness, typically manifests within 24–72 h after surgery and may persist for several days to weeks (3). POD is a frequent and significant complication in older patients having abdominal surgery (4), with reported incidence rates as high as 50% (5). POD is associated with extended hospital stays, a rise in postoperative complication risks, and considerably higher healthcare costs. (6). Despite its clinical importance, the underlying pathophysiological mechanisms of POD remain poorly elucidated (7). Moreover, evidence supporting effective pharmacological prevention strategies is limited (8). Therefore, early identification, accurate prediction, and timely intervention are critical to improving clinical outcomes in these patients.

Cognitive reserve, which is closely associated with brain function, is increasingly recognized as a potential risk factor for POD. Cognitive reserve represents the brain’s ability to cope with neuropathological damage, and higher educational achievement is believed to enhance this capacity, potentially reducing the risk of POD (9–11). However, the relationship between educational level and POD remains underexplored. Existing studies have primarily focused on clinical and demographic factors, such as age, preoperative cognitive impairment, and comorbidities (12, 13). Most POD prediction do not incorporate educational level as a key variable, which may limit their accuracy and generalizability (14, 15). In particular, there is insufficient evidence about how educational level affects POD in older patients having abdominal surgery.

In summary, this study aims to investigate the association between educational level and the risk of POD in older patients undergoing abdominal surgery by employing a two-sample cohort design, which includes an exploratory cohort and a validation cohort. The findings are expected to provide evidence for the early identification of patients who are at high risk for POD in the clinical older abdominal surgery population.

## 2 Materials and methods

### 2.1 Study design and participants

**FIGURE 1:**
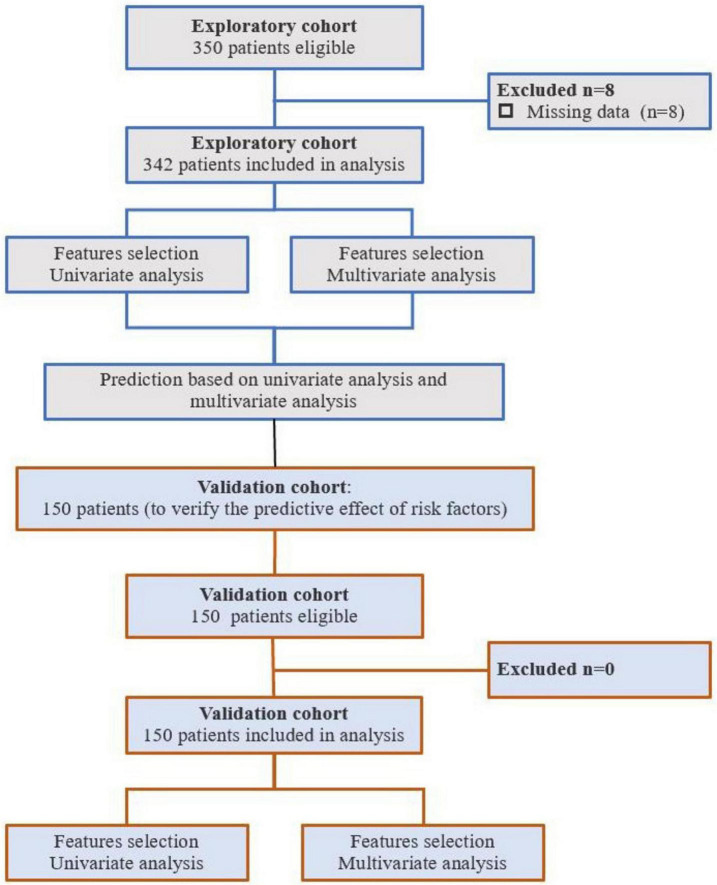
Study flow chart.

As shown in Figure 1, this study prospectively collected clinical data from 492 patients who had abdominal surgery at the Second Affiliated Hospital of Chongqing Medical University between August 2022 and July 2023. Eligibility criteria were: ① age range of 65–90 years; ② patients scheduled for elective surgery under general anesthesia; ③ patients scheduled for elective abdominal procedures; and ④ American Society of Anesthesiologists physical status classification of I to III. Exclusion criteria comprised the following: ① patients with known psychiatric disorders, communication difficulties, or cognitive impairments (preoperative Mini-Mental State Examination (MMSE) score < 24) prior to surgery; ② patients with severe hearing, visual, or speech impairments that hindered communication with the investigators; ③ patients on long-term sedatives, antidepressants, or with a history of alcohol abuse; and ④ patients who refused or were unable to complete cognitive assessments. The allocation principle was applied for analysis and validation. The inclusion and exclusion process for patients is demonstrated in Figure 1. The study was authorized by the Ethics Committee of the Second Affiliated Hospital of Chongqing Medical University (Approval No.: 2024-33).

### 2.2 Data collection

General information, including gender, age, educational level, American Society of Anesthesiologists classification, history of allergies, preoperative smoking status, preoperative alcohol consumption, and postoperative complications, were collected. Preoperative biochemical indicators such as hemoglobin concentration, serum protein concentration, absolute neutrophil count, and absolute white blood cell count were recorded. In addition, surgical type and major abdominal surgeries with a duration exceeding 2 h were analyzed.

### 2.3 Assessment methods

#### 2.3.1 Preoperative emotional assessment

• The West China Mood Index was used to evaluate preoperative emotional status.

#### 2.3.2 POD assessment (primary outcome measures)

• From postoperative days 1 to 3, trained investigators assessed the occurrence of POD using the 3D-CAM (3-Minute Diagnostic Interview for Confusion Assessment Method), with follow-ups conducted twice daily.
• Patients with at least one positive POD result within the first three postoperative days were considered to have developed POD and were assigned to the POD group.
• Patients without any positive POD results were included in the non-POD group.

#### 2.3.3 Postoperative assessment in the PACU (post-anesthesia care unit) (secondary outcome measures)

• Sedation status was assessed using the Richmond Agitation-Sedation Scale.
• Delirium during the recovery period was evaluated using the Confusion Assessment Method for the Intensive Care Unit.
• Patients with a positive Confusion Assessment Method for the Intensive Care Unit result during the recovery period were diagnosed with PACU POD and included in the PACU POD group.
• Patients without delirium were included in the non- PACU POD group.

### 2.4 Risk factor scoring and validation

A cohort study involving 342 patients was selected as the exploratory cohort, and an additional 150 patients meeting the same criteria were included as the validation cohort. Based on the information from the exploratory cohort and the presence or absence of delirium, relevant risk factors were identified. Logistic regression was used to determine the link between these variables and the risk of POD.

### 2.5 Sample size

According to the principle of 10 events per variable (EVP), eight factors including age, BMI, education level, ASA, surgery time, anesthesia time, surgical type and hemoglobin were included, and considering a 5% loss to follow-up rate, 25% (16) the incidence of postoperative delirium in elderly patients undergoing gastric and colorectal surgery under general anesthesia, the exploratory cohort plans to include 337 patients. The validation cohort mainly considers the factor of educational level and a sample size of 150 cases in the validation cohort is sufficient.

### 2.6 Statistical analysis

Statistical Methods: Continuous variables were summarized according to their distributional properties. Variables following normal distribution were expressed as mean ± standard deviation, whereas those deviating from normality were reported as median (interquartile range, IQR). For intergroup comparisons, independent samples *t*-tests were used for normally distributed continuous variables, while the Mann–Whitney U test was applied for non-normally distributed variables. Categorical variables were expressed as frequencies and percentages, and group comparisons were performed using the chi-square test or Fisher’s exact test, as appropriate. To confirm the inclusion of potential predictors connected to the outcome, variables showing a *P*-value of less than 0.1 in the univariate logistic regression analysis were added as covariates in the multivariate logistic regression to pinpoint independent risk factors. Using SPSS 26 (IBM Corp, USA) and R 4.4.0 (R Foundation for Statistical Computing, Austria), all statistical analyses were carried out, considering a two-tailed *P*-value below 0.05 as statistically significant.

## 3 Results

### 3.1 Basic characteristics

This study included two cohorts comprising 350 and 150 patients, respectively. Based on predefined exclusion criteria, 8 (missing data) and 0 patients were excluded from each group, resulting in 342 patients in the exploratory set and 150 patients in the validation set. Table 1 provides a detailed comparison of demographic and clinical variables among the exploratory set and validation set. There is no statistical difference in the main characteristics between the two cohorts.

**TABLE 1:** Basic characteristics of all patients.

Variables	Exploratory cohort (*n* = 342)	Validation cohort (*n* = 150)	*P*-value
Age, median (IQR)	71 (68, 75)	71 (67, 75)	0.91
Female	130 (38)	59 (39.3)	0.781
BMI, median (IQR)	22.86 (20.43, 25.09)	23.02 (20.86, 25.14)	0.608
Education level			0.572
Primary school or below	165 (48.2)	75 (50)	
Middle school	121 (35.4)	56 (37.3)	
College	56 (16.4)	19 (12.7)	
ASA, n (%)			<0.001
ASA I	20 (5.8)	21 (14)	
ASA II	172 (50.3)	89 (59.3)	
ASA ≥ III	150 (43.9)	40 (26.7)	
Disease history	252 (73.7)	110 (73.3)	0.935
HEI, median (IQR)	0 (0, 2)	0.5 (0, 2)	0.67
Hemoglobin, median (IQR)	122 (108, 134)	118 (108, 131)	0.147
WBC (×10^∧^9/L), median (IQR)	5.73 (4.5, 7.32)	5.7 (4.57, 7.42)	0.895
NC (×10^∧^9/L), median (IQR)	3.8 (2.8, 5.05)	3.92 (2.8, 5.12)	0.854
LC (×10^∧^9/L), median (IQR)	1.25 (0.87, 1.55)	1.25 (0.84, 1.53)	0.936
MC (×10^∧^9/L), median (IQR)	0.39 (0.29, 0.52)	0.39 (0.29, 0.51)	0.711
PLT (×10^∧^9/L), median (IQR)	211 (165, 266)	203.5 (155.75, 264)	0.592
NLR, median (IQR)	3.16 (2.16, 4.52)	3 (2.3, 4.79)	0.764
dNLR, median (IQR)	0.87 (0.83, 0.9)	0.87 (0.83, 0.9)	0.984
MLR, median (IQR)	0.32 (0.23, 0.47)	0.32 (0.24, 0.48)	0.948
nMLR, median (IQR)	3.48 (2.47, 4.92)	3.34 (2.51, 5.2)	0.851
SIRI, median (IQR)	1.19 (0.71, 2)	1.16 (0.72, 2.28)	0.828
Surgery time > 4 h	119 (34.8)	57 (38)	0.495
Anesthesia time > 4 h	149 (43.6)	79 (52.7)	0.062
PCIA	312 (91.2)	134 (89.3)	0.506
PACU POD	124 (36.3)	50 (33.3)	0.532
Postoperative POD at 3 days	65 (19)	50 (33.3)	0.001

Data are presented as *n* (%) and median (IQR). IQR, interquartile range; BMI, body mass index; ASA, American Society of Anesthesiologists; HEI, Huaxi emotional-distress index; WBC, white blood cell count; NC, neutrophil count; LC, lymphocyte count; MC, monocyte count; PLT, platelet count; NLR, neutrophil-to-lymphocyte ratio; dNLR, derived neutrophil-to-lymphocyte ratio; MLR, monocyte-to-lymphocyte ratio; nMLR, neutrophil-to-monocyte ratio; SIRI, systemic inflammation response index; PCIA, patient-controlled intravenous analgesia; PACU, post-anesthesia care unit; POD, postoperative delirium.

The median age of patients in both the exploratory and validation sets was 71 years (exploratory cohort: IQR 68–75 years; validation cohort: IQR 67–75 years). The proportions of females were 38.0 and 39.3% in the exploratory and validation cohort, respectively. The median body mass index was 22.86 kg/m^2^ (IQR 20.43–25.09) and 23.02 kg/m^2^ (IQR 20.86–25.14) in the exploratory and validation cohort, respectively. Analysis of educational levels revealed similar proportions of patients with middle school and college education in both cohorts (exploratory cohort: 48.2 and 35.4%; validation cohort: 50.0 and 37.3%).

The exploratory cohort had 73.7% of patients with a history of comorbidities, compared to 73.3% in the validation cohort. In the exploratory cohort, 36.3% of patients experienced postoperative delirium (POD) in the post-anesthesia care unit (PACU), compared to 33.3% in the validation cohort. The overall incidence of POD within the first 3 days after surgery was 19.0 and 33%, respectively. Additional detailed characteristics of the cohorts are summarized in Table 1.

### 3.2 Univariate logistic regression analysis for POD at 3 days and PACU POD

In the exploratory cohort, univariate logistic regression analysis (Table 2) revealed several variables significantly associated with postoperative delirium (POD) at 3 days. An increase in age was linked to a higher risk of POD (OR = 1.06, 95% CI: 1.01–1.11, *P* = 0.028). Conversely, elevated hemoglobin levels were associated with a reduced risk of POD (OR = 0.94, 95% CI: 0.89–0.99, *P* = 0.028). Compared to participants with primary education or less, those with a college education demonstrated a lower risk of POD (OR = 0.99, 95% CI: 0.97–1.00, *P* = 0.019). Prolonged surgical duration exceeding 4 h was significantly associated with an elevated risk of POD (OR = 2.90, 95% CI: 1.67–5.04, *P* < 0.001), as was anesthesia duration longer than 4 h (OR = 2.28, 95% CI: 1.31–3.95, *P* = 0.003).

**TABLE 2:** Univariable logistic regression analysis of risk factors for POD at 3 days.

Characteristics	Exploratory cohort		Validation cohort	
	OR (95% CI)	*P*-value	OR (95% CI)	*P*-value
Age	1.06 (1.01–1.11)	0.028	1.03 (0.97–1.1)	0.342
Female	1.2 (0.69–2.08)	0.515	0.81 (0.4–1.63)	0.555
BMI	0.94 (0.87–1.02)	0.16	0.95 (0.86–1.06)	0.376
**Education level**				
Primary school or below	Reference		Reference	
Middle school	0.64 (0.35–1.17)	0.144	0.45 (0.21–0.96)	0.038
College	0.39 (0.15–0.97)	0.043	0.36 (0.11–1.18)	0.092
**ASA**				
ASA I			Reference	
ASA II	0.51 (0.18–1.44)	0.205	0.83 (0.3–2.28)	0.711
ASA ≥ III	0.54 (0.19–1.52)	0.24	1.48 (0.49–4.46)	0.487
Disease history	1.12 (0.6–2.08)	0.729	1.45 (0.65–3.21)	0.362
HEI	0.96 (0.82–1.13)	0.633	1.05 (0.92–1.19)	0.474
Hemoglobin	0.99 (0.97–1)	0.019	1 (0.98–1.01)	0.737
WBC	1.04 (0.93–1.15)	0.524	1.09 (0.96–1.25)	0.182
NC	1 (0.95–1.06)	0.923	1 (0.94–1.06)	0.921
LC	0.83 (0.51–1.37)	0.467	0.87 (0.46–1.62)	0.655
MC	0.89 (0.29–2.75)	0.843	1.33 (0.44–3.98)	0.615
PLT	1 (1–1)	0.318	1 (1–1.01)	0.255
NLR	1 (0.95–1.05)	0.958	1.02 (0.95–1.1)	0.547
dNLR	1.02 (0.85–1.24)	0.797	0.68 (0.13–3.45)	0.639
MLR	1.08 (0.48–2.43)	0.847	1.57 (0.57–4.34)	0.382
nMLR	1 (0.96–1.05)	0.95	1.02 (0.95–1.1)	0.516
SIRI	0.99 (0.92–1.08)	0.883	1.01 (0.93–1.11)	0.79
Surgery time > 4h	2.9 (1.67–5.04)	<0.001	3.55 (1.74–7.23)	<0.001
Anesthesia time > 4h	2.28 (1.31–3.95)	0.003	2.28 (1.13–4.61)	0.022
PCIA	2.23 (0.66–7.6)	0.199	3.907 (0.85–17.91)	0.079

POD, postoperative delirium; BMI, body mass index; ASA, American Society of Anesthesiologists; HEI, Huaxi emotional-distress index; WBC, white blood cell count; NC, neutrophil count; LC, lymphocyte count; MC, monocyte count; PLT, platelet count; NLR, neutrophil-to-lymphocyte ratio; dNLR, derived neutrophil-to-lymphocyte ratio; MLR, monocyte-to-lymphocyte ratio; nMLR, neutrophil-to-monocyte ratio; SIRI, systemic inflammation response index; PCIA, patient-controlled intravenous analgesia.

In the validation cohort, univariate logistic regression analysis (Table 2) demonstrated significant associations with postoperative delirium (POD) at 3 days. Participants with a college education exhibited a reduced risk of POD compared to those with primary education or less (OR = 0.45, 95% CI: 0.21–0.96, *P* = 0.0389). Prolonged surgical duration exceeding 4 h was associated with an elevated risk of POD (OR = 3.55, 95% CI: 1.74–7.23, *P* < 0.001), as was anesthesia duration longer than 4 h (OR = 2.28, 95% CI: 1.13–4.61, *P* = 0.022). These findings corroborate the significant relationships between education level, surgical duration, anesthesia duration, and the risk of POD at 3 days, as previously observed in the exploratory cohort.

In addition to POD at 3 days, this study also explored risk factors for POD in the PACU (Table 3). In the univariate logistic regression analysis of the exploratory cohort data, the following results were observed: compared to individuals with primary school or lower education level, those with a college education had a lower risk of PACU POD (OR = 0.49, 95% CI: 0.25–0.96, *P* = 0.038); surgical time greater than 4 h was significantly associated with an increased risk of PACU POD (OR = 2.03, 95% CI: 1.28–3.21, *P* = 0.003); anesthesia time greater than 4 h was also associated with an increased risk of PACU POD (OR = 1.85, 95% CI: 1.18–2.89, *P* = 0.007).

**TABLE 3:** Univariable logistic regression analysis of risk factors for PACU POD.

	Exploratory cohort		Validation cohort	
Characteristics	OR (95% CI)	*P*-value	OR (95% CI)	*P*-value
Age	1.03 (0.98–1.07)	0.249	1 (0.93–1.07)	0.982
Female	1.17 (0.74–1.83)	0.507	0.62 (0.31–1.27)	0.195
BMI	1 (0.99–1.01)	0.933	0.98 (0.88–1.09)	0.727
**Education Level**				
Primary school or below	Reference		Reference	
Middle school	0.81 (0.5–1.31)	0.384	0.47 (0.22–1.01)	0.053
College	0.49 (0.25–0.96)	0.038	0.51 (0.17–1.55)	0.234
**ASA**				
ASA I	Reference		Reference	
ASA II	1.73 (0.6–5)	0.308	1.55 (0.52–4.64)	0.436
ASA ≥ III	1.79 (0.62–5.18)	0.285	2.13 (0.65–7)	0.211
Disease history	1.11 (0.67–1.84)	0.677	1.23 (0.56–2.69)	0.602
HEI	0.92 (0.8–1.05)	0.221	0.85 (0.68–1.07)	0.178
Hemoglobin	1 (0.99–1.01)	0.704	1.01 (1–1.03)	0.103
WBC	0.95 (0.86–1.04)	0.272	0.97 (0.84–1.11)	0.654
NC	0.95 (0.87–1.04)	0.305	0.94 (0.81–1.09)	0.407
LC	0.9 (0.61–1.34)	0.607	1.05 (0.57–1.94)	0.873
MC	1.34 (0.56–3.2)	0.507	1.78 (0.56–5.64)	0.325
PLT	1 (1–1)	0.289	1 (1–1)	0.751
NLR	0.97 (0.92–1.02)	0.294	0.92 (0.82–1.04)	0.192
dNLR	0.76 (0.48–1.21)	0.251	0.01 (0–2.9)	0.114
MLR	1.32 (0.68–2.56)	0.409	1.39 (0.51–3.77)	0.519
nMLR	0.98 (0.93–1.02)	0.33	0.94 (0.84–1.04)	0.222
SIRI	0.99 (0.93–1.06)	0.822	1 (0.91–1.1)	0.983
Surgery time > 4h	2.03 (1.28–3.21)	0.003	1.87 (0.94–3.75)	0.076
Anesthesia time > 4h	1.85 (1.18–2.89)	0.007	2.97 (1.44–6.12)	0.003
PCIA	1.36 (0.6–3.07)	0.457	2.341 (0.64–8.63)	0.201

POD, postoperative delirium; BMI, body mass index; ASA, American Society of Anesthesiologists; HEI, Huaxi emotional-distress index; WBC, white blood cell count; NC, neutrophil count; LC, lymphocyte count; MC, monocyte count; PLT, platelet count; NLR, neutrophil-to-lymphocyte ratio; dNLR, derived neutrophil-to-lymphocyte ratio; MLR, monocyte-to-lymphocyte ratio; nMLR, neutrophil-to-monocyte ratio; SIRI, systemic inflammation response index; PCIA, patient-controlled intravenous analgesia.

In the validation cohort, univariate logistic regression analysis (Table 3) identified significant associations between prolonged surgical and anesthesia durations and an increased risk of PACU POD. Surgical time exceeding 4 h showed a trend toward higher risk (OR = 1.87, 95% CI: 0.94–3.75, *P* = 0.076), while anesthesia time greater than 4 h was significantly associated with elevated risk (OR = 2.97, 95% CI: 1.44–6.12, *P* = 0.003). Although higher education levels did not achieve statistical significance, a trend toward reduced PACU POD risk was observed (middle school: OR = 0.47, 95% CI: 0.22–1.01, *P* = 0.053; college: OR = 0.51, 95% CI: 0.17–1.55, *P* = 0.234).

### 3.3 Multivariate logistic regression analysis for POD at 3 days and PACU POD

**FIGURE 2:**
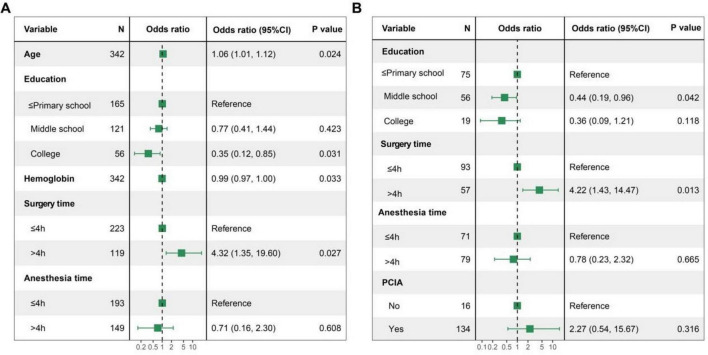
Forest plots for POD at 3 days in two cohorts. **(A)** Multivariate logistic regression analysis for POD at 3 days in the exploratory cohort; **(B)** multivariate logistic regression analysis for POD at 3 days in the validation cohort. POD, postoperative delirium.

In this study, variables exhibiting a *P*-value below 0.1 in the univariate logistic regression analysis were incorporated into the multivariate logistic regression. Forest plots were subsequently generated to visualize the outcomes for both cohorts. In addition, collinearity analysis was performed before multivariate logistic regression analysis, and the variance inflation factor between all variables was less than 2. The analysis results showed that in the exploratory cohort, age, college education level, hemoglobin level, and surgical duration were independently associated with POD at 3 days (Figure 2A). Specifically, age was positively correlated with POD at 3 days (OR = 1.06, 95% CI: 1.01–1.12, *P* = 0.024); although middle school education did not reach statistical significance, a trend toward a reduced risk of POD at 3 days was observed (OR = 0.64, 95% CI: 0.35–1.17, *P* = 0.144). College education was negatively correlated with POD at 3 days (OR = 0.35, 95% CI: 0.13–0.91, *P* = 0.031); higher hemoglobin levels were negatively correlated with POD at 3 days (OR = 0.99, 95% CI: 0.97–1.00, *P* = 0.033); and a surgical time greater than 4 h significantly increased the risk of POD at 3 days (OR = 4.32, 95% CI: 1.18–15.82, *P* = 0.027).

In the validation cohort, education level remained independently associated with POD at 3 days (Figure 2B). Specifically, secondary education showed a significant relationship with POD at 3 days (OR = 0.44, 95% CI: 0.2–0.97, *P* = 0.042). Although college education did not achieve statistical significance, a trend toward a reduced risk of POD at 3 days was observed (OR = 0.36, 95% CI: 0.1–1.29, *P* = 0.118). Surgical durations exceeding 4 h remained significantly associated with an elevated risk of POD at 3 days (OR = 4.22, 95% CI: 1.18–15.82, *P* = 0.013). Furthermore, the use of patient-controlled intravenous analgesia was also linked to an increased risk of POD at 3 days, although this association did not reach statistical significance (OR = 2.27, 95% CI: 0.46–11.29, *P* = 0.316).

**FIGURE 3:**
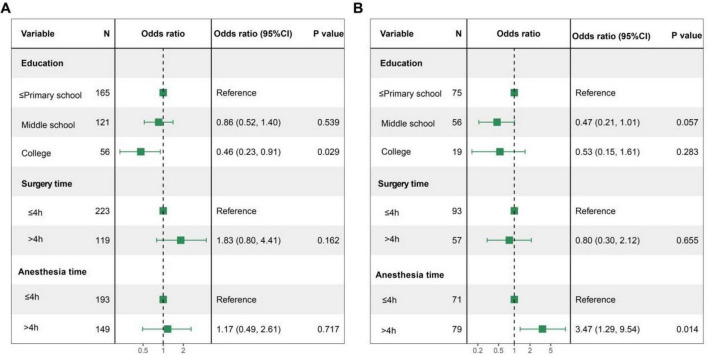
Forest plots for PACU POD in two cohorts. **(A)** Multivariate logistic regression analysis for PACU POD in the exploratory cohort; **(B)** multivariate logistic regression analysis for PACU POD in the validation cohort. PACU, post-anesthesia care unit; POD, postoperative delirium.

This study further analyzed the independent risk factors for PACU POD. In the exploratory cohort, multivariate logistic regression analysis showed that education level was independently associated with PACU POD (Figure 3A). The relationship between secondary education and PACU POD was not statistically significant (OR = 0.86, 95% CI: 0.52–1.40, *P* = 0.539), while college education was significantly associated with PACU POD (OR = 0.46, 95% CI: 0.23–0.92, *P* = 0.029).

In the validation cohort, compared to those with primary school or lower education levels, those with middle school and college education levels showed a trend toward reducing the risk of PACU POD. However, the result did not reach statistical significance. For secondary education, a trend toward a reduced risk of PACU POD was observed, though it did not reach statistical significance (OR = 0.47, 95% CI: 0.21–1.02, *P* = 0.057). Similarly, college education showed a non-significant trend toward risk reduction (OR = 0.53, 95% CI: 0.17–1.69, *P* = 0.283). In contrast, anesthesia durations exceeding 4 h were significantly associated with an elevated risk of PACU POD (OR = 3.47, 95% CI: 1.29–9.35, *P* = 0.014). These results are presented in Figure 3B.

## 4 Discussion

This study, through two cohort studies, identified and validated that university education level, surgical duration, and anesthesia time are independently associated with POD within 3 days after surgery. Although there were differences in the incidence of postoperative delirium between the exploration cohort and the validation cohort, education level was confirmed to be an independent risk factor in both cohorts, and its influence degree was higher than other related factors. This finding suggests that education level as a risk factor for postoperative delirium has universal applicability across populations and should be paid special attention in clinical practice. Lower educational attainment was associated with a significantly increased risk of POD within 3 days postoperatively, while surgical or anesthesia durations exceeding 4 h also significantly increased the risk of POD. Additionally, extended anesthesia time (>4 h) was independently correlated with the development of PACU POD. The findings emphasize the significance of accounting for educational attainment and surgical or anesthesia duration when assessing and managing the risk of POD.

Previous studies have predominantly investigated contributors to POD risk in older abdominal surgery patients using single-center retrospective or prospective cohort designs (17–22). In contrast to previous research, this study innovatively employed a dual independent cohort design, comprising an exploratory cohort and a validation cohort, to systematically analyze and validate risk factors for POD. Furthermore, the study categorized POD into two critical phases—PACU POD and POD within 3 days and independently validated risk factors for each phase. This study employed a dual-assessment strategy for postoperative delirium detection, utilizing the non-verbal-adapted CAM-ICU (23) for immediate post-anesthesia evaluation in sedated or mechanically ventilated patients, followed by the verbal-based 3D-CAM (24) for subsequent assessments as cognitive function recovered, ensuring comprehensive delirium subtype identification throughout the perioperative recovery continuum. This phased research design not only enhances the reliability of the findings but also comprehensively covers the entire course of POD occurrence, providing more robust evidence for clinical prevention and management.

Lower educational attainment was found to be a significant predictor of POD in this study, likely attributable to the protective role of cognitive reserve, which tends to be more robust in those with advanced educational attainment. These individuals are likely better equipped to comprehend and adhere to preoperative and postoperative medical instructions, thereby potentially mitigating the risk of POD. This finding emphasizes the need to integrate educational background into delirium risk assessment and management, enabling tailored patient education and support strategies to optimize outcomes (25). In addition, patients with higher education usually have a broader social support network and can obtain more emotional and practical support after surgery (26–28) so as to reduce the risk of postoperative delirium. Some previous studies on the risk factors of POD may have ignored the influence of education level. This omission may be due to the limitations of the study design, or the association between education level and POD has not been fully revealed. However, as a potential socio-economic factor, education level may play an important role in the mechanism of POD (10).

This study identified lower educational attainment as a significant risk factor for POD during the resuscitation phase in the exploratory cohort. Also, it showed a trend of increasing the risk of delirium during resuscitation in the confirmatory cohort. This phenomenon may be related to the cumulative damage of neurons, dendrites, receptors, and microglia in older patients, making them more prone to delirium under biological stress (29). The compensatory ability for neuroinflammation and blood-brain barrier dysfunction induced by surgery and anesthesia is weak. The release of proinflammatory cytokines and markers of nerve injury can damage large-scale neuronal networks, leading to acute cognitive dysfunction (30–32), eventually triggering the destruction of large-scale neuronal networks in the brain, which leads to acute cognitive dysfunction (33). Individuals with high cognitive reserve usually have more abundant neural pathways to cope with various cognitive challenges. According to the neural network theory, the neural network formed by synaptic connections between neurons is not static but has high plasticity, which can continuously develop and reorganize with the accumulation of individual learning and experience (11, 34). This neural plasticity is considered to be one of the important mechanisms for individuals with high cognitive reserve to show stronger adaptability in the face of neurodegenerative diseases or brain injury (35). However, the exact association between education level and POD risk and its potential mechanism still needs to be further verified and clarified through large-scale, multicenter, prospective studies in order to provide a more reliable evidence-based basis for clinical intervention.

This study also found that with the increase in age, the risk of POD increased significantly 3 days after the operation, a finding consistent with previous research (33). Previous studies have repeatedly established age as a significant independent predictor of adverse outcomes following various types of surgeries (1). This may be caused by brain tissue degeneration and changes in central neurotransmitters in older patients (36, 37). In addition, this study also found that the operation time and anesthesia time were related to POD. Prolonging anesthesia time meant that more fluid input, more complex surgery, more challenging intraoperative conditions, and the use of a variety of drugs were needed, which were related to the occurrence of POD (38).

This study still has some limitations. First, the sample sizes of the two independent cohorts in this study are relatively small, which may restrict the generalizability of the research findings. In the future, larger scale multi-center studies should be carried out to construct and verify POD models using the risk factors identified in this study, and to improve the external validity of the results. Furthermore, the single-center design of the current study may introduce potential selection bias. To address this limitation, future research should adopt a multicenter approach, which would significantly improve the external validity and broader applicability of the findings. Second, potential confounding factors, such as type of surgery; depth of anesthesia; socio-economic status and pre-existing cognitive impairment, are not fully adjusted, which may affect the observed association. Third, the level of education is widely classified, and more detailed stratification can provide a better understanding of the relationship between education and POD. Finally, the follow-up period was 3 days after surgery, and the post operative cognitive decline dysfunction for a longer time after surgery was not evaluated. Cases of delayed or persistent delirium may be ignored. Future studies should address these limitations to further clarify the risk factors of postoperative delirium.

In brief, a lower education level is a risk factor for POD 3 days after the operation, and operation or anesthesia time of more than 4 h also significantly increases the risk of POD. These findings emphasize the importance of education level in reducing the incidence of POD in preoperative assessment and postoperative care. It is necessary to conduct further research to explore the potential mechanism and formulate targeted intervention measures for high-risk cohorts. It is recommended to further investigate the role of education level as a significant risk factor for postoperative delirium (POD). Multicenter studies with larger sample sizes should be conducted to validate and expand upon these findings.

## Data Availability

The raw data supporting the conclusions of this article will be made available by the authors, without undue reservation.

## References

[1] Luo Y Wu X Song Y Wang X Liu K Shi C Development and validation of a nomogram to predict postoperative delirium in older patients after major abdominal surgery: A retrospective case-control study. *Perioper Med (Lond).* (2024) 13:41. 10.1186/s13741-024-00399-3 38755693 PMC11100071

[2] Wilson J Mart M Cunningham C Shehabi Y Girard T MacLullich A Delirium. *Nat Rev Dis Primers.* (2020) 6:90. 10.1038/s41572-020-00223-4 33184265 PMC9012267

[3] Silverstein J Timberger M Reich D Uysal S. Central nervous system dysfunction after noncardiac surgery and anesthesia in the elderly. *Anesthesiology.* (2007) 106:622–8. 10.1097/00000542-200703000-00026 17325520

[4] Bhattacharya B Maung A Barre K Maerz L Rodriguez-Davalos M Schilsky M Postoperative delirium is associated with increased intensive care unit and hospital length of stays after liver transplantation. *J Surg Res.* (2017) 207:223–8. 10.1016/j.jss.2016.08.084 27979481

[5] Hughes C Boncyk C Culley D Fleisher L Leung J McDonagh D American society for enhanced recovery and perioperative quality initiative joint consensus statement on postoperative delirium prevention. *Anesth Analg.* (2020) 130:1572–90. 10.1213/ANE.0000000000004641 32022748 PMC7379173

[6] He X Shi X Wang Y Han S Liu J Yang F Genetic assessment of the causal effect of plasma metabolites and metabolic pathways on delirium. *Anesthesiol Perioperative Sci.* (2024) 2:28. 10.1007/s44254-024-00064-4

[7] Jin Z Hu J Ma D. Postoperative delirium: Perioperative assessment, risk reduction, and management. *Br J Anaesth.* (2020) 125:492–504. 10.1016/j.bja.2020.06.063 32798069

[8] Duning T Ilting-Reuke K Beckhuis M Oswald D. Postoperative delirium – Treatment and prevention. *Curr Opin Anaesthesiol.* (2021) 34:27–32. 10.1097/ACO.0000000000000939 33315641

[9] O’Hanlon S Baxter M Hosie A. Postoperative delirium in older patients with cancer: The role of psychological distress and social support. *Curr Opin Support Palliat Care.* (2022) 16:38–47. 10.1097/SPC.0000000000000588 34939608

[10] Feinkohl I. Post-operative cognitive impairment: A cognitive epidemiology perspective. *J Intell.* (2022) 10:18. 10.3390/jintelligence10010018 35324574 PMC8949407

[11] Stern Y. Cognitive reserve in ageing and Alzheimer’s disease. *Lancet Neurol.* (2012) 11:1006–12. 10.1016/S1474-4422(12)70191-6 23079557 PMC3507991

[12] Ahn E Bang S. Risk factors associated with treatment of hyperactive postoperative delirium in elderly patients following hip fracture surgery under regional anesthesia: A nationwide population-based study. *Braz J Anesthesiol.* (2022) 72:213–9. 10.1016/j.bjane.2021.03.020 33915191 PMC9373072

[13] O’Sullivan R Inouye S Meagher D. Delirium and depression: Inter-relationship and clinical overlap in elderly people. *Lancet Psychiatry* (2014) 1:303–11. 10.1016/S2215-0366(14)70281-0 26360863 PMC5338740

[14] Shi Z Mei X Li C Chen Y Zheng H Wu Y Postoperative delirium is associated with long-term decline in activities of daily living. *Anesthesiology.* (2019) 131:492–500. 10.1097/ALN.0000000000002849 31335550 PMC6692194

[15] van Meenen L van Meenen D de Rooij S ter Riet G. Risk prediction models for postoperative delirium: A systematic review and meta-analysis. *J Am Geriatr Soc* (2014) 62:2383–90. 10.1111/jgs.13138 25516034

[16] Morimoto Y Yoshimura M Utada K Setoyama K Matsumoto M Sakabe T Prediction of postoperative delirium after abdominal surgery in the elderly. *J Anesth.* (2009) 23:51–6. 10.1007/s00540-008-0688-1 19234823

[17] Bosancic Z Spies C Müller A Winterer G Piper S Heinrich M Association of cholinesterase activities and POD in older adult abdominal surgical patients. *BMC Anesthesiol.* (2022) 22:293. 10.1186/s12871-022-01826-y 36114455 PMC9479414

[18] Liu J Li J He J Zhang H Liu M Rong J. The Age-adjusted Charlson comorbidity Index predicts post-operative delirium in the elderly following thoracic and abdominal surgery: A prospective observational cohort study. *Front Aging Neurosci.* (2022) 14:979119. 10.3389/fnagi.2022.979119 36062155 PMC9428551

[19] Lai C Liu K Tsai C Hsu J Hsueh S Hung C Risk factors and effect of postoperative delirium on adverse surgical outcomes in older adults after elective abdominal cancer surgery in Taiwan. *Asian J Surg.* (2023) 46:1199–206. 10.1016/j.asjsur.2022.08.079 36041906

[20] Janssen T Steyerberg E Faes M Wijsman J Gobardhan P Ho G Risk factors for postoperative delirium after elective major abdominal surgery in elderly patients: A cohort study. *Int J Surg.* (2019) 71:29–35. 10.1016/j.ijsu.2019.09.011 31526896

[21] Olin K Eriksdotter-Jönhagen M Jansson A Herrington M Kristiansson M Permert J. Postoperative delirium in elderly patients after major abdominal surgery. *Br J Surg.* (2005) 92:1559–64. 10.1002/bjs.5053 16231283

[22] Ehrlich A Oh E Psoter K Bettick D Wang N Gearhart S Incidence of post-operative delirium increases as severity of frailty increases. *Age Ageing.* (2024) 53:afae168. 10.1093/ageing/afae168 39148434 PMC11327404

[23] Ely EW Inouye SK Bernard GR Gordon S Francis J May L Delirium in mechanically ventilated patients: Validity and reliability of the confusion assessment method for the intensive care unit (CAM-ICU). *JAMA.* (2001) 286:2703–10. 10.1001/jama.286.21.2703 11730446

[24] Kuczmarska A Ngo LH Guess J O’Connor MA Branford-White L Palihnich K Detection of delirium in hospitalized older general medicine patients: A comparison of the 3D-CAM and CAM-ICU. *J Gen Intern Med.* (2016) 31:297–303. 10.1007/s11606-015-3514-0 26443577 PMC4762827

[25] Tow A Holtzer R Wang C Sharan A Kim S Gladstein A Cognitive reserve and postoperative delirium in older adults. *J Am Geriatr Soc.* (2016) 64:1341–6. 10.1111/jgs.14130 27321616 PMC4916859

[26] Featherstone I Hosie A Siddiqi N Grassau P Bush S Taylor J The experience of delirium in palliative care settings for patients, family, clinicians and volunteers: A qualitative systematic review and thematic synthesis. *Palliat Med.* (2021) 35:988–1004. 10.1177/02692163211006313 33784915 PMC8189008

[27] Arias F Chen F Fong T Shiff H Alegria M Marcantonio E Neighborhood-level social disadvantage and risk of delirium following major surgery. *J Am Geriatr Soc.* (2020) 68:2863–71. 10.1111/jgs.16782 32865254 PMC7744425

[28] Do T Lemogne C Journois D Safran D Consoli S. Low social support is associated with an increased risk of postoperative delirium. *J Clin Anesth.* (2012) 24:126–32. 10.1016/j.jclinane.2011.07.002 22301203

[29] Rump K Adamzik M. Epigenetic mechanisms of postoperative cognitive impairment induced by anesthesia and neuroinflammation. *Cells.* (2022) 11:2954. 10.3390/cells11192954 36230916 PMC9563723

[30] Xu F Chen H Gao Y Yang X Zhang C Ni X. Sodium butyrate ameliorates postoperative delirium by regulating gut microbiota dysbiosis to inhibit astrocyte activation in aged mice. *Neurochem Res* (2024) 49:3342–55. 10.1007/s11064-024-04245-2 39340594

[31] Hu Y Hu X He Z Liu Y Gui Y Zhu S Anesthesia/surgery activate MMP9 leading to blood-brain barrier disruption, triggering neuroinflammation and POD-like behavior in aged mice. *Int Immunopharmacol.* (2024) 135:112290. 10.1016/j.intimp.2024.112290 38796964

[32] Capogna E Watne L Sørensen Ø Guichelaar CJ Idland AV Halaas NB Associations of neuroinflammatory IL-6 and IL-8 with brain atrophy, memory decline, and core AD biomarkers – in cognitively unimpaired older adults. *Brain Behav Immun.* (2023) 113:56–65. 10.1016/j.bbi.2023.06.027 37400002

[33] Inouye S Westendorp R Saczynski J. Delirium in elderly people. *Lancet.* (2014) 383:911–22. 10.1016/S0140-6736(13)60688-1 23992774 PMC4120864

[34] Park D Reuter-Lorenz P. The adaptive brain: aging and neurocognitive scaffolding. *Annu Rev Psychol.* (2009) 60:173–96. 10.1146/annurev.psych.59.103006.093656 19035823 PMC3359129

[35] Barulli D Stern Y. Efficiency, capacity, compensation, maintenance, plasticity: Emerging concepts in cognitive reserve. *Trends Cogn Sci.* (2013) 17:502–9. 10.1016/j.tics.2013.08.012 24018144 PMC3840716

[36] Thedim M Vacas S. Anesthetic sensitivity and resilience in the aging brain: Implications for perioperative neurocognitive disorders. *Anesthesiol Perioperative Sci.* (2025) 3:11. 10.1007/s44254-025-00094-6

[37] Tune L Damlouji N Holland A Gardner T Folstein M Coyle J. Association of postoperative delirium with raised serum levels of anticholinergic drugs. *Lancet.* (1981) 2:651–3. 10.1016/s0140-6736(81)90994-6 6116042

[38] Brown C LaFlam A Max L Wyrobek J Neufeld K Kebaish K Delirium after spine surgery in older adults: Incidence, risk factors, and outcomes. *J Am Geriatr Soc.* (2016) 64:2101–8. 10.1111/jgs.14434 27696373 PMC5407286

